# Quantitative Trait Loci for Phenology, Yield, and Phosphorus Use Efficiency in Cowpea

**DOI:** 10.3390/genes16010064

**Published:** 2025-01-08

**Authors:** Saba B. Mohammed, Patrick Obia Ongom, Nouhoun Belko, Muhammad L. Umar, María Muñoz-Amatriaín, Bao-Lam Huynh, Abou Togola, Muhammad F. Ishiyaku, Ousmane Boukar

**Affiliations:** 1 International Institute of Tropical Agriculture, PMB 3112, Kano 700223, Nigeria; s.mohammed@cgiar.org (S.B.M.); n.belko@cgiar.org (N.B.); a.togola@cgiar.org (A.T.); o.boukar@cgiar.org (O.B.); 2 Department of Plant Science, Ahmadu Bello University, PMB 1044, Zaria 810211, Nigeria; mlumar@abu.edu.ng (M.L.U.); mfishiyaku@abu.edu.ng (M.F.I.); 3 Africa Rice Center (AfricaRice), 01 B.P. 2551, Bouake 01, Côte d’Ivoire; 4 Department of Botany and Plant Sciences, University of California, Riverside, CA 94607, USA; mmuna@unileon.es; 5 Departamento de Biología Molecular (Área Genética), Universidad de León, 24071 León, Spain; 6 Department of Nematology, University of California, 900 University Avenue, Riverside, CA 92521, USA; baolamh@ucr.edu; 7 International Maize and Wheat Improvement Center, World Agroforestry Centre Campus, UN Avenue Gigiri, Nairobi P.O. Box 1041-00621, Kenya

**Keywords:** cowpea, phosphorus use efficiency, low soil phosphorus, grain yield, quantitative trait loci, soil fertility

## Abstract

Background/Objectives: Cowpea is an important legume crop in sub-Saharan Africa (SSA) and beyond. However, access to phosphorus (P), a critical element for plant growth and development, is a significant constraint in SSA. Thus, it is essential to have high P-use efficiency varieties to achieve increased yields in environments where little-to- no phosphate fertilizers are applied. Methods: In this study, crop phenology, yield, and grain P efficiency traits were assessed in two recombinant inbred line (RIL) populations across ten environments under high- and low-P soil conditions to identify traits’ response to different soil P levels and associated quantitative trait loci (QTLs). Single-environment (SEA) and multi-environment (MEA) QTL analyses were conducted for days to flowering (DTF), days to maturity (DTM), biomass yield (BYLD), grain yield (GYLD), grain P-use efficiency (gPUE) and grain P-uptake efficiency (gPUpE). Results: Phenotypic data indicated significant variation among the RILs, and inadequate soil P had a negative impact on flowering, maturity, and yield traits. A total of 40 QTLs were identified by SEA, with most explaining greater than 10% of the phenotypic variance, indicating that many major-effect QTLs contributed to the genetic component of these traits. Similarly, MEA identified 23 QTLs associated with DTF, DTM, GYLD, and gPUpE under high- and low-P environments. Thirty percent (12/40) of the QTLs identified by SEA were also found by MEA, and some of those were identified in more than one P environment, highlighting their potential in breeding programs targeting PUE. QTLs on chromosomes Vu03 and Vu08 exhibited consistent effects under both high- and low-P conditions. In addition, candidate genes underlying the QTL regions were identified. Conclusions: This study lays the foundation for molecular breeding for PUE and contributes to understanding the genetic basis of cowpea response in different soil P conditions. Some of the identified genomic loci, many being novel QTLs, could be deployed in marker-aided selection and fine mapping of candidate genes.

## 1. Introduction

Cowpea (*Vigna unguiculata* L. Walp) is a crucial food and nutrition security crop for several millions of people in sub-Saharan Africa (SSA) and elsewhere, due to its high content of some essential amino acids naturally low in cereal grains [1,2,3]. Global production is estimated at 8.9 million tons per annum on over 14 million ha, with a large proportion coming from smallholder farmers in SSA [4]. The crop’s yields are typically low in farmers’ fields, due to a combination of biotic and abiotic constraints. Poor soil fertility, especially nitrogen (N), phosphorus (P) and organic matter content, is a key edaphic limitation to the cowpea’s productivity, especially in the sandy soils of SSA. Phosphorus is required for optimum symbiotic N fixation, early maturity, optimal growth, and productivity [5,6,7]. Cowpea can fix a considerable amount of N through biological N fixation only when there is adequate P level in the soil, indicating that the crop’s ability to fix N is greatly limited in soils with low plant-available soil P [5,6,8]. Over 50% of the global arable land is P deficient [9,10], including soils of most cowpea-producing areas [11,12,13]. The deficiency of P in most crops may be attributed to low total-soil P and, in most cases, low plant-available P [14,15]. There is a substantial difference between the former and latter, as most soils contain a significant amount of P, but the element is often immobile and bound to the upper strata of the soil, thereby making plants access only a small fraction of the total soil P [16,17]. Due to the formation of phosphate complexes, the element is often held in unavailable forms by Al and Fe in acidic soils and by Ca in alkaline soils [18,19]. Whilst P is the second most important macro-nutrient after N for cultivated crop plants, many farmers are not even aware of its importance or cannot afford P-fertilizers, and, therefore, low plant-available soil P can be synonymous with hidden hunger for cowpea [20]. For soils with low total-P content, applying P-fertilizers to soils appears to be a quick fix, but this increases crop production costs, accelerates the depletion of non-renewable P resources, and can result in environmental problems associated with P run-off to water bodies. Therefore, developing varieties with improved P efficiency is desirable for sustainable agriculture [15,21,22], whereas for soils with low plant-available P, the common strategy is to improve P uptake efficiency and internal use efficiencies [14,15]. The amount of P available in soil solution for plant uptake is conditioned by several factors such as soil type, pH, root association with arbuscular mycorrhiza fungi (AMF), root architecture, and genotype of the crop [17,23,24]. Some findings have associated higher biomass and grain yield of cowpea in low plant-available P soils with improved root architecture, association with AMF and exudation of organic compounds [25,26].

Indicators for P use and acquisition efficiency in cowpea include high shoot dry weight (biomass), root dry biomass, shoot-to-root ratio, P concentration in shoot and root, grain yield, early flowering, and maturity [7,27,28] and computed P efficiency traits such as P-use efficiency, uptake and P efficiency ratio [14,29,30]. Several screening studies have revealed genetic variation for adaptation to low soil P and response to applied P, indicating the possibility of developing varieties for different soil P conditions [7,30,31]. Among several parameters often investigated for P acquisition ability, P uptake is an integrative trait that directly reflects a plant’s ability to acquire P, which has been used to identify a major quantitative trait locus (QTL) named *Pup1* in rice [32]. This QTL has proven effective, as lines carrying *Pup1* increase P uptake in a severely P-deficient field, and the higher yields of modern rice varieties are partly attributed to improved P use efficiency for grain yield [33,34]. In cowpea, there are few reports on QTLs and markers for P efficiency traits, especially using high-density SNP markers. This has partly limited the capacity to deploy markers in the selection of P-efficient lines in breeding to increase precision and reduce the time taken to achieve genetic gains in developing P-efficient varieties. Few SSR markers and QTLs for P-use efficiency using shoot dry biomass and tissue P concentration have been reported [34]. In addition, ten SNP markers were found to be associated with adaptation to low P and response to rock phosphate through a genome-wide association study [27]. However, these few studies were conducted in single growing seasons, and no QTLs have been detected so far in different years at the same or different locations, or in different genetic populations using high-density SNP maps. As most P-related traits are influenced by many loci and often exhibit significant genotype-by-environment interaction, QTLs that are stable across years, environments or different genetic backgrounds can be used to improve P efficiency [14], necessitating the need to investigate QTLs association with key performance traits in different environments or seasons, and genetic backgrounds.

Preliminary phenotyping by our team showed that cowpea lines IT84S-2246 and Yacine are performing well in low-P soil conditions and respond positively to applied P, whereas TVu-14676 and 58–77 have contrasting performance under high- and low-P conditions [35]. The RIL populations used in this work derive from these contrasting parents (TVu-14676 x ×IT84S-2246 and Yacine ×58-77) and have been previously used by different groups to map QTLs for nematode and *Striga* resistance and yield components [36,37,38]. These two populations were evaluated under high and low soil-P conditions representing common conditions of most farmers’ fields across five locations in Nigeria between 2017 and 2018. Phenology, biomass yield, grain yield and P efficiency traits were evaluated to investigate the response of the RILs to varying soil P treatments and to identify QTLs associated with performance traits in cowpea. This study will foster further breeding work to design and implement marker-aided selection targeting the development of varieties with high yield potential in low-P soils and an improved response to applied-P fertilizers.

## 2. Materials and Methods

### 2.1. Plant Materials

Two biparental populations of RILs derived from TVu-14676 × IT84S-2246 (“TV × IT”, thereafter), with 130 RILs, and Yacine × 58-77 (“YA × 58”, thereafter), composed of 95 RILs, refs. [38,39,40] were evaluated, together with their parents and three checks for each population. All RILs from the TV × IT population were used for QTL mapping, whereas only 84 lines were used for marker-trait associations in the YA × 58 population, due to high levels of missing genotypic data. The RILs had been advanced by single seed descent (SSD) to at least F9 [36]. Pure seeds from each RIL and parents were provided by the cowpea research team at the University of California Riverside (UCR), USA. The seeds were treated with Bayer Thiram and accompanied by a Phytosanitary Certificate for shipment to Africa. The TVu-14676 and IT84S-2246 parents were from IITA’s worldwide collection [38], whereas Yacine and 58-77 were both from Senegal [38], and are characterized by brown and black smooth seed coats, respectively. Muchero et al. [41], reported that TV × IT segregated for *Striga* and nematode resistance, and YA × 58 segregated for flower thrip resistance and individual grain weight. In addition, our previous screening experiments indicated that TVu-14676, IT84S-2246, Yacine and 58-77 had contrasting performances for biomass and grain yield under high and low soil-P conditions using both nutrient–sand solution and the natural field environment [35].

### 2.2. Phenotyping Sites and Experimental Design

Field evaluation of the RIL populations was carried out across ten unique environments of high-P (HP) and low-P (LP) soil at Zaria-2017, Zaria-2018, Minjibir-2018, Kadawa-2018 and Mokwa-2018, covering three savanna agro-ecologies i.e., northern Guinea, Sudan, and southern Guinea Savanna zones in Nigeria. These P conditions mimic the typical P fertility levels in optimized experimental fields (HP) and most smallholder farmers’ fields (LP). The specific environments were as follows: Institute for Agricultural Research (IAR) low-P field, Zaria (N11009′49.6″ E007037′13.8″) in 2017 and 2018 wet seasons, the low-P plot of the International Institute of Tropical Agriculture (IITA) at Minjibir farm (N12°0′8.464″, E008°3′9.971″) in 2018 dry season, IAR Irrigation Research Station, Kadawa (N11058′.844″ E008033′.451″) in 2017 dry season, and IAR Agricultural Research Station, Mokwa (N9021′8.892″, E00501′23.874″) in 2018 wet season, each under high- and low-soil conditions. The average plant-available soil-P contents and other physical and chemical properties of the fields measured at 0–20 cm depth before planting ranged from low to moderate, measuring 6.5 mg kg^−1^ for Zaria, 7.8 mg kg^−1^ for Mokwa, and 10.8 mg kg^−1^ for Kadawa, all Bray I P.

Before planting, seeds were treated with a commercial fungicide (AllStar) at 10 g 4 kg^−1^ of seeds, according to the manufacturer’s recommendation. Ten seeds per RIL line were received and planted in pots in a screen house to increase the stock for the initial 2017 experiment in Zaria, and subsequent experiments used seeds harvested from the first experiment. Phosphorus (P) was applied to high-P (HP) plots using a commercial single super phosphate (SSP) fertilizer at the rate of 60 kg P_2_O_5_ ha^−1^ seven days after sowing (DAS). The SSP used contained 20% total P_2_O_5_, 15% water-soluble P_2_O_5_, 16.5% water and citrate-soluble P_2_O_5_, 11% Sulphur, 18% Ca and 4% moisture (TAK-Agro SSP 20%). Two single fertilizers, urea (N-46%) and muriate of potash (MOP-K_2_O 60%), were applied at the rate of 30 kg ha^−1^ for both HP and LP plots to avoid potential confounding effects of N and K_2_O. The LP plots did not receive any SSP fertilizer, and the plants’ performance in the LP plots was sustained on the inherent soil P. All the single fertilisers were applied by placing them below the soil surface approximately 5 cm away from the plant hills, five DAS. Each trial was laid in 15 × 9 and 10 × 10 alpha lattice designs for TV × IT and YA × 58 populations, respectively, each with three replications. Each RIL and parents were planted 20 cm apart in a single 2 m row plot with 75 cm between adjacent plots. Approximately 20–30 seeds were planted per plot in 10 hills, with each hill containing 2–3 seeds initially, but later thinned down to one plant per hill, giving a total of ten plants per row. Plants were protected against insect pests by spraying insecticide (Karate 50 g/L lambda-cyhalothrin, Syngenta Crop Protection AG, Basel, Switzerland), and crop management (ploughing, weeding, and pest control) across environments, following local recommended practices [42,43].

### 2.3. Phenotypic Data Collection and Analysis

Plant traits assessed in this study were grouped into three categories: phenology (DTF and DTM), yield (BYLD and GYLD), and P efficiency (gPUE and gPUpE) measured at crop maturity. The DTF and DTM were counted as the number of days from sowing to when 10% of the plants had the first flower and had attained 95% maturity, respectively (Table S1). Biomass yield was measured in grams as the dry weight of two plants sampled before flowering, whereas grain yield was measured as the dry weight of grains in kilograms per hectare, as described by Mohammed et al. [44]. When all measurements were taken, harvesting was carried out on a plot basis into individually labelled bags, pods were threshed, and seeds were weighed. To measure the P-efficiency traits of the grains, dried grain samples were ground to a fine powder using a grinding mill, and 1 g of each sample was weighed out using a digital balance and packed into small Ziplock bags. These samples were sent to the Phosphorus laboratory of the Department of Soil Science, Ahmadu Bello University Nigeria, to determine P concentration using the Vanado-molybdate (Yellow) procedure [45]. The total grain P content of the lines was calculated as a product of grain P concentration and grain dry weight per line, whereas the gPUE and gPUpE were calculated as described previously [7,15] using the following formulae.

$$Phosphorus\;use\;efficiency\;(grain)=\frac{Total\;grain\;weight\;at\;maturity\;(kg)}{Total\;grain\;P\;nutrient\;at\;maturity\;(g)},$$

whereas the

$$Phosphorus\;uptake\;efficiency\;(grain)=\frac{Total\;grain\;P\;nutrient\;at\;maturity\;(Nt)}{P\;nutrient\;supplied\;(Ns)}$$

where the Nt = grain weight at maturity (kg/ha) × P nutrient concentration in the grain (%) and Ns = P nutrient applied to the soil. The phenotypic data were analysed using the linear mixed model to generate means of RIL lines over replications. For each trait, the best linear unbiased estimate (BLUE) and best linear unbiased prediction (BLUP) were calculated using the multi-environmental trial analysis (META-R) software [46], while traits correlation analysis was conducted to identify the important pattern of relationship between them under HP and LP conditions.

### 2.4. Genotypic Data and Genetic Linkage Map Construction

The genotypic data and genetic maps for the two RIL populations were sourced from the cowpea team at UC Riverside, USA, with that of the TV × IT population now being publicly available from Muñoz-Amatriaín et al. [40], while data of the YA × 58 population are provided in Table S2. The procedure used for the genotyping is briefly described here. Genomic DNA from each RIL line and parents were extracted from dried young seedling leaves using the Plant DNeasy procedure and quantified with a Quant-IT dsDNA kit. The detailed explanation for DNA extraction, quantification and purity check has been described previously [40]. The RIL lines were genotyped at the University of Southern California (USA) using the Illumina Cowpea iSelect Consortium Array containing 51,128 SNPs. Prior to linkage map construction, lines with high heterozygosity and those carrying nonparental alleles were removed. Only SNPs that were polymorphic between the parents and the RILs and had minor allele frequencies > 0.20 were used for the map construction in MSTmap (http://www.mstmap.org/, accessed on 5 November 2024). Specific MSTmap parameters used for each population are described in Muñoz-Amatriaín et al. [40] and Steinbrenner et al. [47]. The linkage groups were numbered and oriented according to cowpea pseudomolecules [48]. The TV × IT genetic map has 14,660 SNPs [40], whereas there were 17,638 SNPs for the YA × 58 genetic map across the 11 linkage groups (Table S2).

### 2.5. QTL Mapping for Phenology, Yield, and Phosphorus Efficiency Traits

We used two approaches for conducting single-environment QTL analysis (SEA): Rqtl [49] and QTL IciMapping [50]. The Rqtl analysis was performed following the procedure described by Broman et al. [49] and implemented in R following Herniter et al. [51], with few modifications (Appendix A). The Rqtl enabled the use of all the SNPs in the single-environment QTL analysis, while QTL IciMapping utilized one SNP per genetic bin for both the single- and multiple-environment QTL identifications, resulting in a total of 1216 SNPs with 111, 82, 186, 81, 126, 119, 105, 131, 126, 67 and 82, respectively, on chromosomes 1 through 11 in the TV × IT population. There was a total of 1244 SNPs in the YA × 58 with 94, 84, 193, 99, 124, 85, 141, 100, 136, 83 and 105 on chromosomes 1 through 11, respectively. One SNP per bin was used in IciMapping analysis because there were many SNPs mapped to the same position on the chromosomes, and the software does not process such co-segregating SNPs, as it is deemed not to provide additional information. However, it is essential to consider all markers within a genetic bin for a proper estimation of the physical size and location of the QTLs. SNPs in the format of A, B, X, and “U” representing the genotype for parent 1 and 2, and heterozygous and missing data were converted to the numeric (2, 0, 1 and −1), respectively, in MS Excel for further use in the QTL IciMapping. All significant QTLs identified by Rqtl were also detected by IciMapping, except a few significant SEA QTL loci exclusively identified by IciMapping, which are denoted with asterisks in the tables.

In the R package “qtl”, the “read.cross” function linked phenotype and genotype files, while “jittermap” reassigned SNP positions to handle SNPs with identical cM locations. Genotype probabilities were estimated with “cal.genoprob”, and QTL loci were mapped using “scanone” with the EM algorithm and Haley–Knott (HK) regression, both producing similar results. QTL significance was tested using 1000 permutations on the HK output. The “fitqtl” function, after creating a QTL object with “makeqtl”, was used to estimate the proportion of phenotypic variance explained by the identified QTLs. Confidence intervals for QTL locations were calculated using “lodint” and the argument “expandtomarkers = True” was used to expand the intervals to identify the nearest flanking markers, whereas, to use the QTL IciMapping, genotypic and phenotypic data were integrated for additive and dominant QTLs (ICIM-ADD) according to the software’s procedure [50]; these are widely used in QTL mapping studies [3,52,53,54,55]. The IciMapping was performed by fitting additive genetic effects in the genome scan using two steps, stepwise regression to select significant markers (*p* < 0.001) associated with phenotypes, then adjusting phenotypic values for the selected markers except for the two flanking markers and using the adjusted phenotypic values in composite interval mapping, which tests the QTL additive effect of the QTLs and epistatic interaction between them [56]. The LOD threshold was determined with a 1000 PT, and the mapping parameters were set as a window scan step of 1 cM and PIN = 0.001. The stepwise regression determined the phenotypic variance explained (PVE%) by individual QTL, additive, and dominance effects at LOD peaks [57]. The stability of the QTLs across environments [50,58,59] was assessed using the multi-environment QTL analysis module of QTL IciMapping v4.2, using similar parameters as SEA. A similar QTL nomenclature system described by Lo et al. [60] was adopted with little modification, where “c” indicates cowpea followed by the abbreviation of the trait, soil-P condition, and the chromosome number, and sometimes with an ordered number when more than one QTL is detected in a single chromosome for a trait. In addition, “ME” precedes QTLs identified from multi-environment QTL analysis, e.g., *CByH1.3* indicates the third QTL associated with cowpea biomass yield under high soil P on chromosome 1. MapChart version 2.32 software was used to display QTLs on chromosomes [61]. To display the loci on a common framework for both populations, the positions of the markers flanking the QTL regions were mapped onto a consensus genetic map [40], using the new chromosome numbering (María Muñoz-Amatriaín, 2024, personal communication; see Table S3).

### 2.6. Identification of Candidate Genes

To identify candidate genes associated with significant loci for the individual traits, SNP positions in the reference genome of cowpea IT97K-499-35v1.2 [48,62] were identified, and annotated genes within QTL regions downloaded from Phytozome (https://phytozome-next.jgi.doe.gov/info/Vunguiculata_v1_2, accessed on 5 November 2024) and the Legume Information System (https://data.legumeinfo.org/Vigna/unguiculata/, accessed on 5 November 2024) were used. Candidate genes within the significant QTL regions whose function was related to traits of interest were identified, and their function was searched in the literature.

## 3. Results

### 3.1. Phenotypic Evaluation of the RILs for Crop Phenology, Yield and P Efficiency Traits in Varying Soil-P Conditions

**Figure 1:**
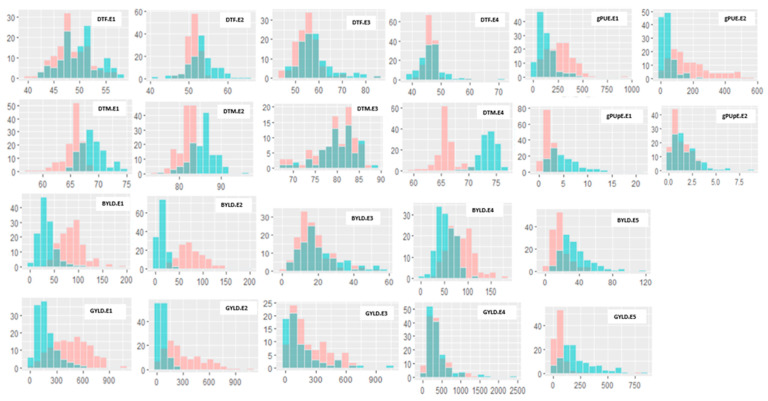
Frequency distributions of phenotypic data under high-and low-P environments in the TVu-14676 x IT84S-2246 population. High P is depicted in red colour, low P in sky blue, and blue-grey is the overlap between environments. The horizontal and vertical axes represent the trait value and number of genotypes, respectively. DTF.E1–E4 = days to flowering for Environment 1 to 4, DTM.E1–E4 = days to maturity for Environment 1 to 4, BYLD.E1–E5 = biomass yield for Environment 1 to 5, GYLD.E1–E5 = grain yield (kg/ha) for Environment 1–5, gPUE.E1–E2 = P-use efficiency for Environment 1 and 2, and gPUpE.E1–E2 = P-uptake efficiency of grain for Environment 1 and 2. E1 = 2017.Zaria, E2 = 2018.Zaria, E3 = 2018.Minjibir, E4 = 2018.Mokwa and E5 = 2018.Kadawa.

**Figure 2:**
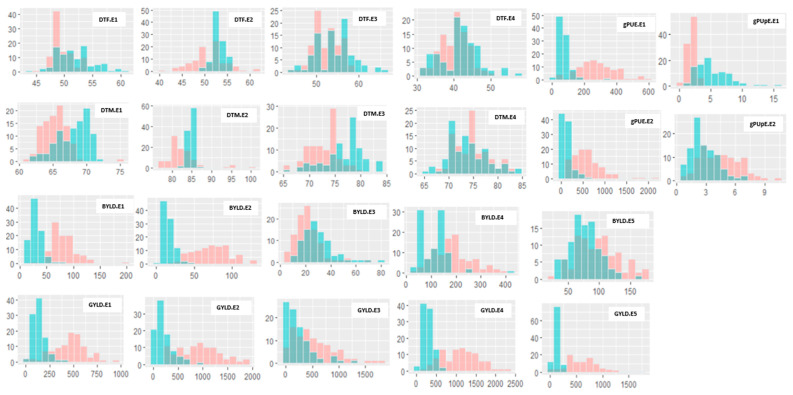
Frequency distributions of phenotypic data under high-P and low-P environments in the Yacine × 58-77 population. High P is illustrated in red colour, low P in sky blue, and blue-grey is the overlap between environments. The horizontal and vertical axes represent the trait value and number of genotypes, respectively. DTF.E1–4 = days to flowering for Environment 1 to 4, DTM.E1–4 = days to maturity for Environment 1 to 4, BYLD.E1–5 = biomass yield for Environment 1 to 5, GYLD.E1–5 = grain yield (kg/ha) for Environment 1–5, gPUE.E1–2 = P-use efficiency for Environment 1 and 2 and gPUpE.E1–2 = P-uptake efficiency for Environment 1 and 2. E1 = 2017.Zaria, E2 = 2018.Zaria, E3 = 2018.Minjibir, E4 = 2018.Mokwa and E5 = 2018.Kadawa.

Crop plant phenology, yield components and phosphorus efficiency traits significantly varied among the RILs, and trait values were consistently superior under HP as compared to LP conditions (Figure 1 and Figure 2, Tables S4 and S5). This suggested that superior performance depends on the trait type with lower values for DTF and DTM, and higher values for biomass, grain yield, and grain P efficiency. However, it is worth noting that certain lines exhibited higher performance under LP conditions, highlighting the existence of genotypic variation within the populations. Likewise, some trait values of the RILs were observed to exceed the parent lines in both directions, suggesting transgressive segregation, which indicates that both parents contributed favourable alleles for these traits (Tables S4 and S5). The trait distributions were relatively continuous among the RILs for many traits under both P conditions and populations (Figure 1 and Figure 2), suggesting a quantitative inheritance and the suitability of these populations for QTL mapping.

In both RIL populations, an increase in P supply from LP to HP had notable effects on flowering and maturity, resulting in earlier events under HP conditions, compared to slight delays under LP conditions. Furthermore, increased biomass, grain, and phosphorus use efficiency for grain (gPUE) were observed as the soil-P condition changed from LP to HP, signifying a reduction in performance under LP conditions for most of the traits in both individuals (Table S4) and across the locations (Table S5). Interestingly, the gPUpE was found to be higher under LP conditions across most environments. The two parents for each of the populations had contrasting performances for nearly all traits across the two P conditions, especially under the LP conditions. Specifically, IT84S-2246 was superior to Tvu-14676 for most traits in the TV × IT population, whereas Yacine had higher values for biomass and grain yield over 58-77 in the YA × 58 population. The parental lines maintained near mean values for DTF under LP conditions. The combined BLUP analysis for the two soil-P levels across the environments revealed significant genotype and genotype-by-environment (G × E) interactions in both populations (*p* < 0.0001 or *p* < 0.05) for most traits (Table S6). The exceptions were DTM and gPUE under the HP condition in the TV × IT population and biomass, and gPUE and gPUpE under the LP condition for the YA × 58 population. The broad-sense heritability (H^2^) for both RIL populations in combined environments ranged from 20 to 50% for gPUpE and DTF in HP and 10 to 60% for grain yield and DTF in LP for the TV × IT population, whereas it varied from 20 to 60% for DTF and gPUE under HP and 10 to 40% for gPUpE and DTF under LP for the YA × 58 population, indicating low-to-moderate heritabilities for the traits measured (Table S6). Similarly, the correlation analysis for both combined and individual environments demonstrated a significant association between HP and LP for most traits in both populations (Table S7).

### 3.2. QTL Analysis Under Contrasting Soil Phosphorus Conditions

The single-environment QTL analysis (SEA) has identified a total of 40 QTLs. Ten of these QTLs were associated with DTF, nine with DTM, five with BYLD, ten with GYLD, four with gPUE, and two with gPUpE across HP and LP conditions. These QTLs were distributed over nine chromosomes, with the phenotypic variance explained (PVE) ranging between 4.3 and 32.5% (Table 1 and Table 2). The multi-environment QTL analysis (MEA) identified twenty-three QTLs: nine for DTF, two for DTM, eight for GYLD, and four for grain PUpE (Table 3).

#### 3.2.1. Days to Flowering

**Figure 3:**
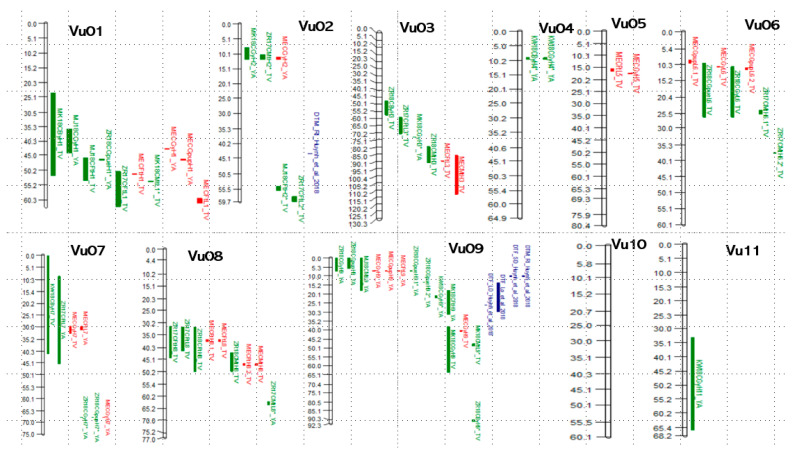
Chromosome-wide distribution of the identified QTLs in comparison with previous studies. QTL regions are shown by chromosome. cM positions are on the left, with some positions skipped. SEA QTLs from the present study are represented in green, MEA QTLs are represented in red, and QTLs from other studies are shown in blue.

Eight QTLs associated with Flowering time (FT) were identified in the TV × IT, with four of these detected under HP conditions. The phenotypic variance explained (PVE), QTL regions and the genes associated with these QTLs have been presented in Table 1, Tables S8 and S9. Consistent flowering QTLs were identified on Vu01 and Vu08 under both P conditions (Figure 3), including those identified through MEA, indicating their stability across different environments. The PVE of these MEA QTLs ranged from 3.0% to 27.3% under HP, whereas under LP, it ranged from 8.0% to 11.7% (Table 3). In the YA × 58 population, two QTLs were associated with FT. The PVE, QTL regions and the related genes are shown in Table 1, Tables S8 and S9. The MEA of this population identified two loci under LP on Vu07 and Vu09, explaining 13.7% and 18.4% of the trait’s phenotypic variance (Table 3, Figure 3). Several flowering-time candidate genes were identified in some of the QTLs using both TV × IT and YA × 58 populations, and these have been presented in Table 4.

#### 3.2.2. Days to Maturity

QTLs for DTM were identified on Vu01, Vu02, Vu03, Vu06, Vu08, and Vu09. Five loci were identified in the TV × IT population under two HP conditions (Table 1). Similarly, two QTLs were identified under the LP condition on Vu01 and Vu09, explaining 10.4% and 9.8% of the phenotypic variance, respectively. In the YA × 58, two QTLs were linked to DTM only under LP; they are _ZR17_CMtL8 and _MJ18_CMtL9 on Vu08 and Vu09, with PVE of 15.2% and 20.0% (Table 1). The regions and genes associated with the QTL f have been provided in Table 1, Tables S8 and S9. DTM QTLs on Vu03 and Vu08 exhibited an MEA effect under HP (Table 3), meaning they are stable across different P environments and can be good targets for improving cowpea maturity traits. The PVE by the MEA loci in the TV × IT population was 4.5–6.6% under HP conditions (Table 3). No MEA QTL was found in LP conditions for this population. Similarly, the YA × 58 MET did not reveal significant loci associated with DTM (Table S10). Among the annotated genes in these regions (Table 4), maturity-related candidate genes were identified. 

#### 3.2.3. Biomass Yield

QTLs for biomass yield were detected on Vu01, Vu03, Vu04, Vu07, and Vu09. Four QTLs were identified in the TV × IT population under HP environments, with two at ZR.2018HP, and one each at MK.2018HP and KW.2018HP; these QTLs had PVE ranging from 10.3% to 12.0% (Table 2). In the YA × 58 population, one significant locus was found at KW.2018HP on Vu04, explaining 18.7% of the phenotypic variance. The annotated genes have been provided in Tables S8 and S9. Minor QTLs explaining between 4.8% and 13.0% of biomass phenotypic variance were identified across both populations (Table S11). Genes involved in biomass accumulation include those involved in cellulose synthesis, photosynthesis, and hormone regulation, and these have been presented in Table 5.

#### 3.2.4. Grain Yield

Grain yield QTLs were identified on Vu01, Vu02, Vu03, Vu04, Vu06, Vu07, Vu09, and Vu11 across the two populations. Specifically, two QTLs were detected for grain yield in the TV × IT population, with one each under HP and LP in MK.2018HP and 2018LP.ZR, explaining 13.2% and 13.6% of the phenotypic variances, respectively (Table 2). In the YA × 58 population, eight loci were associated with grain yield, with two each at ZR.2018HP on Vu07 and Vu09, two at MK.2018HP on Vu02 and Vu03, one at MJ.2018HP on Vu01 and three loci at KW.2018HP on Vu11, Vu04, and Vu09. The grain yield loci explain 10.3% to 32.5% of the observed variation (Table 2). Moreover, other minor QTLs with LOD values below the PT threshold were associated with grain yield under various levels of soil P across both populations, explaining between 6.0% and 13.5% of phenotypic variance (Table S11). The grain yield MEA in the TV × IT revealed the presence of three significant QTLs in HP on Vu05, Vu07 and Vu09, with PVE values that ranged from 6.0 to 18.9% (Table 3). In addition, one QTL was detected on Vu06 under LP, explaining an exceptionally high proportion of the phenotypic variance for the trait. As for the YA × 58 population, four loci were identified under HP with PVE values ranging from 8.8 to 27.2% (Table 3). In contrast, no significant loci were identified under LP in the YA × 58 population, but there were minor QTLs associated with grain yield (Table S11). Annotated genes that play roles in seed development, nutrient transport, flowering time regulation, and stress responses, contributing to improved grain yield, were identified (Table 5).

#### 3.2.5. Grain P-Efficiency Traits

We identified six QTLs associated with grain P-efficiency traits (Table 2). Four loci associated with grain PUE were expressed under HP in 2018LP.ZR, with one on Vu01 and two on Vu09, having a PVE of between 4.5% and 26.9% in the YA × 58 population, where one locus under LP in ZR.2018LPwas also detected on Vu06, accounting for 12.7% of the variation in the TV × IT population. The regions occupied by these QTLs and the annotated genes have been presented in Table 2, Tables S8 and S9. Minor QTLs linked to gPUE under HP and LP conditions in both populations, explaining between 7.3% and 12.0% of the phenotypic variance, were reported (Table S11). Furthermore, two loci were identified for gPUpE at ZR.2018HP on Vu07 and Vu09 with PVE values of 12.7% and 17.8% in the YA × 58 RIL (Table 2). The MEA of TV × IT revealed two QTLs for gPUpE in LP on Vu6.1 and Vu6.2, with PVE values ranging from 1.9% to 4.2%. Equally, the MEA of YA × 58 revealed two QTLs associated with grain PUpE in HP with a PVE value of 26.0% and 25.4% on Vu01 and Vu09 (Table 3), whereas no significant QTLs were identified in LP (Table S11). The candidate genes for grain P efficiency and their functions are described in Table 5.

## 4. Discussion

### 4.1. Response of RILs for Phenology, Yield, and P-Efficiency Traits Under Varying Soil-P Conditions

This study investigated the influence of soil P on the DTF, DTM, BYLD, GYLD, gPUE and gPUpE in two RIL populations with contrasting performance in HP and LP soils. The HP plots mirror conditions in experimental plots where optimum P fertilizers are applied, whereas the LP plots represent farmers’ fields where cowpea is often grown with minimal or no P fertilization. The use of 60 kg ha^−1^ P_2_O_5_ in HP was based on previous work [7,12,63], and to avoid confounding effects of major nutrient deficiencies in the experiment, N and K_2_O were applied through the application of urea and muriate of potash fertilisers. The farmers’ practice of growing cowpea under limited P fertilisation has been documented [13,20,26]. The parental lines of the two RILs exhibited contrasting performances, with IT84S-2246 superior to TVu-14676 (the TV × IT population) and Yacine over 58-77 (the YA × 58 population) for most of the traits under different P soils. The results consistently showed superior performance under HP conditions compared to LP conditions across most environments in both populations, indicating HP conditions provided plants with sufficient P, while LP conditions were suboptimal where yield was negatively impacted. Similar reports have been made [26,35,64]. Some lines exhibited relatively higher performance under LP conditions, indicating genotypic variation and transgressive segregation. The increased performance from LP to HP conditions led to earlier DTF and DTM, and increased BYLD, GYLD, and gPUE. However, there was higher gPUpE under LP conditions across most environments. An indirect effect of P uptake on P-use efficiency in rice evaluated in LP soil has been reported, where lines with high PUE, often having low PUpE, apparently experience extreme P-deficiency stress, and vice versa [32]. Phosphorus-use efficiency can be improved by increasing both P-use and -uptake efficiencies [14,32]. The combined environment BLUP analysis revealed significant genotypic variation and G×E interactions, highlighting the interplay of genetic and environmental factors in determining the performance of key traits. The variation in yield traits and P-efficiency traits in cowpea is consistent with previous studies in rice, brassica, and common beans, where genotypes were evaluated in varying soil-P conditions [14,29,65].

### 4.2. QTLs for Phenology, Yield, and P-Efficiency Traits in Cowpea RIL Populations

The lower arm of Vu01 contains a cluster of QTLs for flowering, maturity, biomass, and grain yield under both HP and LP conditions, including MEA QTLs for DTF, GYLD, and gPUpE, making it a potential target for breeding P-efficient varieties. Vu02 has QTLs associated with DTF, DTM, and GYLD, while Vu03 contains QTLs for phenology traits, BYLD, and GYLD. Vu04 has fewer QTLs, but it does contribute to BYLD and GYLD. On Vu05, MEA QTLs for DTF and GYLD were found, and on Vu06, QTLs related to DTM, GYLD, and grain P efficiencies were identified. Vu07 features important QTLs for DTF, BYLD, GYLD, and grain PUpE. Similarly, Vu08 contains QTLs for flowering and maturity, with some regions consistently detected under HP and LP conditions. Vu09 has many regions associated with DTM, BYLD, GYLD, and P-efficiency traits (Figure 3). The identification of these loci has enhanced our understanding of the genetic basis of these essential agronomic and P-use efficiency traits in cowpea.

#### 4.2.1. QTLs for Days to Flowering and Candidate Genes

Flowering time (FT) is a vital agronomic trait that plays a key role in adapting a variety to specific cropping seasons and environments [60,66] and is associated with important traits such as plant vegetative growth, height, and grain quality [67,68]. Early flowering can serve as a drought escape by allowing genotypes to mature before terminal drought occurs [69,70]. The most stable QTLs for DTF were located on Vu01 and Vu08, with some regions identified consistently across both P environments. The fact that these QTLs were identified in multiple environments suggests that this region plays a crucial role in controlling flowering time, regardless of the soil P level, and makes them valuable targets for breeding programs aiming to develop resilient varieties for P-rich and P-deficient soils. On Vu02, Vu03 and Vu07, QTLs for flowering time were also detected, likely contributing to genetic control of the trait under specific P conditions, as they appear to be more environment-specific and less consistent across P treatments (Figure 3). For QTL outputs to be useful to a breeding program, they need to be stable across environments and/or genetic backgrounds [50,58,59]. The MEA revealed the presence of significant QTLs across P environments, indicating the presence of genetic factors influencing these traits under different conditions [58,59].

QTLs for DTF have been mapped in cowpea using biparental RIL and germplasm populations. For instance, in RIL populations, Lo et al. [60] used SNPs and identified two QTLs on Vu05 and Vu09, explaining 20% and 79.3% of the phenotypic variance. Angira et al. [53] identified QTLs on Vu02 and Vu09 with a PVE of 6.16% and 29.3% [53]. Other studies have reported QTLs on LG1, LG2, and LG7 using SSR markers, with the region on LG1 accounting for 18.5% of the total variance [66,71]. Using RAPD markers, two QTLs for DTF have been reported on LGI and LGIII with PVE of 9.2% and 10.2% [72]. In asparagus (yardlong) bean, a vegetable type of cowpea, significant QTLs were identified on LG10 and LG11 for DTF with PVE of 16% and 31.9% [73]. Additionally, using SSR, ten QTL regions for DTF have been identified on all LGs except LG3 in the yardlong bean [74,75]. The phenotypic variance explained by FT loci in the present study (8.0 to 31.7%) is comparable with previous reports of 5–18.5% [66], 16–30% [75], and 20–79% [60], indicating the influence of multiple genes with small-to-moderate effects [76]. It was not possible to compare all identified QTLs from previous studies, due to the unavailability of the marker sequences and the fact that LGs were not aligned with the reference genome [48]. Similarly, BLAST searches of SSR markers were not possible for Andargie et al. [66,71], since the platform (http://cowpeagenomics.med.virginia.edu/CGKB, accessed on 5 November 2024) hosting SSR sequences is currently not functional. Similarly, FT loci have been reported from GWAS populations, including by Hyunh et al. [77], who assayed the multi-parent advanced generation intercross (MAGIC) population and identified four QTLs on Vu04, Vu05, Vu09 and Vu011 under long-photoperiod environments, explaining between <10% and 31% of the variation. They further mapped loci on Vu01, 04, 05 and Vu09 under a short-photoperiod environment, each explaining <13% of the variance. Olatoye et al. [76] identified 32 QTLs and 42 two-way epistatic interactions underlying FT in MAGIC, with the PVE ranging from 2 to 28% for non-epistatic loci and up to 25% for epistatic loci. Similarly, Ravelombola et al. [78] identified five SNP loci associated with DTI for FT on Vu03 and Vu08. Muñoz-Amatriaín et al. [79] identified 40 significant QTLs associated with DTF under short- and long-day environments, with PVE ranging from 5% to 9%. Paudel et al. [80] reported seven SNPs that explained 8–12% phenotypic variance for the trait.

Few of the significant regions in this study coincided with previously reported QTLs. For instance, the physical base positions of the _MK18_CFtH9 region identified in the YA × 58 (4,959,818–8,386,426 bp) overlap some previously identified FT loci [60,77,79,80], indicating its crucial role in controlling FT in cowpea and consistency across multiple studies. QTL regions on Vu01: _MJ18_CFtH1, _ZR17_CFtL1, _ME_CFtH1, and _ME_CFtL1, were in the same region as the FT locus 2_20430 identified under short-day length [77]. The lower region of Vu01 contains a cluster of QTLs associated with flowering, maturity, biomass, and grain yield under both HP and LP conditions, including MEA QTLs for flowering, grain yield, grain PUE, and PUpE (Figure 3). QTL _MJ18_CFtH2* (32,411,552–32,629,715 bp) with 31.7% PVE was 1.1 Mb upstream of a significant FT locus 2_20613 (31,278,141 bp) detected under the same (Minjibir) short-day environment [79]. A large QTL region _ZR17_CFtL7 (2,161,198–26,683,304 bp) with a PVE of 17.3% overlapped a significant locus 2_11041 (23,575,112 bp) in a short-day environment [79]. Similarly, three significant loci on Vu08 (Figure 3), overlapped the significant locus 2_54333 under short days in Minjibir [79]. In addition, we found that the peak SNP in _ZR17_CFtL1 was 623 Kb upstream of SNP (2_14784) on Vu01 (Figure 3), under a long-day photoperiod environment [79]. These results are generally consistent with polygenic control of flowering time in cowpea.

Photoperiod significantly influences FT, drastically reducing FT under short days compared to long days [76]. Flowering is regulated by a network of genes involved in floral initiation, circadian clock regulation, and photoreception [81]. This study identified many annotated genes underlying FT QTLs under HP and LP environments (Table 4), many of which are homologs to FT genes in *Arabidopsis*, soybean, and other species. In the TV × IT population, five FT regions containing key genes are discussed. QTL–_ZR17_CFtH8, harbour *CONSTANS*-*like* 1–*COL1*, involved in photoperiod sensing and regulation of flowering [82,83]. Its overexpression in *Arabidopsis* accelerates the circadian clock [84]. There was C*OL9*, which delays flowering in *Arabidopsis* when overexpressed [85], and *ELF4*-*like 1*, which is essential for the regulation of FT [81]. A second QTL _ZR18_CFtH8 overlapped the _ZR17_CFtH8 (Figure 3) with additional genes, such as *COL16*, whose overexpression delayed flowering in rice [86]. *UDP-Glycosyltransferase* (*UGT87A2*) regulates flowering time in *Arabidopsis* [87], *COL* isoform X2 is involved in flowering regulation [83], and *Snf1-related protein kinase interactor 1* controls plant carbon metabolism [88]. A third QTL region–_MJ18_CFtH1—harboured a flowering-time control protein *FCA*-like isoform X1 that promotes the transition to flowering in *Arabidopsis* [89] and *Phytochrome A* –*PHY1,* which is responsible for light-induced processes like flowering [90]. Under LP, candidate genes were identified in two QTL regions. QTL _ZR17_CFtL1 contained genes such as *PHY1*, *ELF4*-*like 1*, and a *Kelch repeat F-box* protein, signifying their roles in flowering regulation [81,90]. QTL _ZR17_CFtL8 harboured *COL1*, a known flowering regulator [84]. In the YA × 58 population, QTL region–_MK18_CFtH9 contains *PHYE*, which is involved in the transition from vegetative to reproductive growth [91]. Another QTL–_ZR17_CFtL7—consisted of many FT-related proteins, including *LEA* 25, important for seed maturation [92], *EMBRYONIC FLOWER 2-like isoform X1 and X2,* which are key in repressing the reproductive program [93], *flowering locus protein T,* promoting the transition to flowering [94], *JmjC* domain protein, which prevents premature flowering at elevated temperature in *Arabidopsis* [95], *COL13*, which is important in plant photomorphogenesis [83], and *PHY1*. In addition, several loci containing the *MADS-box* proteins (Table 4) are known to regulate processes such as developing floral organs and promoting flowering [96,97]. Annotated candidate genes with flowering functions, including *FLOWERING LOCUS T*, *MADS-box* protein [79], *UGT87A2, SnF1 kinase* [80] and *Phytochrome E* [60], have been identified in the QTL regions associated with FT in cowpea, indicating multiple genes control the trait, and the need for fine mapping [80].

#### 4.2.2. QTLs for Days to Maturity and Candidate Genes

Early maturity, just like early flowering, is a key phenological attribute [98]. QTLs for maturity were found on Vu01, Vu02, Vu03, Vu06, Vu08, and Vu09. In the TV × IT population, two QTLs for DTM explained between 4.3% and 21.4% of phenotypic variance under HP, while in the YA × 58 population, two QTLs under LP environments explained 15.2% and 20.0% of phenotypic variance. Two QTLs on Vu03 and Vu08 are particularly valuable, due to their MEA effects in the TV × IT population under HP conditions, indicating that they are stable across environments [59,99], making them crucial for practical applications in breeding (121). Identifying various QTLs for DTM across various P environments highlights the quantitative nature of QTLs [76,77,78] and the impact of soil P on the trait. Previous studies have mapped QTLs for DTM using different marker systems. For instance, two QTLs for maturity under optimum conditions were mapped on LGs 07 and 08, explaining between 14.4–28.9% and 11.7–25.2%, respectively, using AFLP markers [41,100]. Similarly, with RAPD markers, five QTLs were identified on LG I with PVE of 8.0% to 12.2% [72], and six QTLs in asparagus bean using SSR markers on LGs 1, 2, 3, 4, 6 and 7 [74]. Few of the QTLs identified in this study overlap with those reported in some previous studies that used a similar marker system and chromosome numbering (Figure 3), suggesting many are novel and further reflect the different conditions under which the plants were assessed. In the TV × IT, QTL _ZR17_CMtH2* (18,317,339–19,125,369 bp) is some 10Mb downstream of a maturity locus 2_10022 under restricted irrigation [77]. Additionally, some FT loci are also associated with MT (Figure 3), suggesting a possible genetic basis for the positive relationship between the two phenological traits [76,77]. In YA × 58 population, QTL region _MJ18_CMtL9 (238,281 to 4,959,818 bp) overlapped the significant maturity loci under restricted irrigation [77]. This same _MJ18_CMtL9 overlapped two previously reported FT regions (432,162 bp) and (972,787 bp) under short- and long-day environments, respectively [79]. Interestingly, MT region _ZR17_CMtH6.1* (21,927,476 to 22,481,486 bp) was 1.4 Mb downstream of the FT region (20,504,149 bp) under a short-day environment [79]. Two QTLs (>10% PVE) were detected on Vu08 in different P environments and populations–_ZR18_CMtH8 and _ZR17_CMtL8* (Figure 3)—suggesting certain genomic regions may play essential roles in maturity, regardless of soil P levels. Understanding trait genetic architecture is paramount for cowpea breeding programs, as a selection of specific QTLs for early or delayed maturity under different P conditions can contribute to developing varieties targeting diverse agro-ecological environments, especially since early-maturing varieties that perform well can boost the crop’s productivity and support the deployment of climate-resilient varieties that can escape droughts, while late-maturing varieties with extended vegetative periods would be desirable for higher fodder yield [80], especially in areas with longer wet seasons.

In the TV × IT population, three regions—_ZR18_CMtH3, _ZR18_CMtH8, and _MK18_CMtL9—harboured annotated genes with flowering and maturity-related functions. Specifically, QTL _ZR18_CMtH3 contained the *COL5* protein that affects flowering time and expression of *FLOWERING LOCUS T* and *SUPPRESSOR OF OVEREXPRESSION* OF *CO1* in *Arabidopsis* [101] and the *LEA-25* protein that is involved in seed maturation and protection of cells from stress during desiccation [92]. QTL _ZR18_CMtH8 contained MT-related genes, including *COL9* isoform X4, which plays a role in the photoperiodic control of flowering [85], the *COL16* protein involved in the regulation of flowering time [102], *COL isoform X2 and X1,* which contribute to the regulation of flowering though light-dependent mechanisms [83], and an *Snf1-related kinase interactor 1* involved in energy homeostasis and stress response [88], while QTL _MK18_CMtL9 has a cluster of three loci encoding *flowering promoting factor 1* (*FPF1*), which regulates flowering through the gibberellin signalling pathway [103]. Additionally, there are the *ELF4-like 3* proteins, whose overexpression can decrease the transcription levels of *CONSTANS* and *FLOWERING LOCUS T* [81], and *MADS-box* transcription factors, which play roles in flower development [104,105].

#### 4.2.3. QTLs for Biomass Yield and Candidate Genes

Biomass yield is a key trait in cowpea [106], especially where haulms are used to feed livestock [107,108]. It is genetically complex, linked to grain yield, and influenced by G×E interaction, requiring extensive testing [108]. While each of the biomass QTLs identified in the present study accounted for a substantial amount of the phenotypic variance, indicating these loci were involved in regulating biomass production, none were identified in multiple environments, suggesting that their expression may vary, depending on soil P levels. This implies that these QTLs are more environment-specific and may require further validation before use in breeding programs aiming to enhance biomass yield across diverse P environments. There are few reports on QTL mapping for biomass production in cowpea. Muchero et al. [106] mapped QTLs for biomass yield under drought stress and identified four loci associated with biomass yield under drought with the significant SNPs located on LG 1, 2, 4 and 7, which are now Vu05, Vu07, Vu03 and Vu02, respectively, on the cowpea consensus genetic map [40]. In Muchero et al. [106], the SNP 1_0589 on LG2–Vu07 (5,320,099 bp), was within a large region we identified as _KW18_CByH7 (203,081 to 24,873,914 bp). Similarly, the SNP 1_0296 on LG4–Vu03 (49,715,358 bp) was some 11 Mb downstream of the end of the _ZR18_CByH3 (15,070,882 to 21,079,537 bp). In addition, two QTLs were identified for dry fodder weight (biomass) on Vu04 under a short-day environment by Munoz-Amatriain et al. [79], with one of them (SNP 2_05693; 3,403,521 bp) being 984 Kb downstream of the end of _KW18_CByH4* (2,220,918 to 2,418,796 bp), identified in the current study. Another study involving RILs assessed under different soil-P conditions identified QTLs related to shoot dry matter using three SSR markers [34], but marker sequences of this study are not accessible to enable comparison of the two studies. Interestingly, most QTLs detected under HP had PVE values above 10%, which indicates that the genetic determinants of high biomass production in favourable P conditions are well-defined, and their manipulation can lead to significant improvements. This probably suggests why many studies utilized shoot dry weight (biomass yield) as a criterion for assessing responses to P treatments [7,28,109]. Though all the loci identified in LP environments did not meet the significant threshold, we observed that most of these minor QTLs had PVE values ranging from 5.0% up to 13.0% (Table S7), highlighting the genetic complexity of biomass-yield response to limited soil P.

Genes involved in cell wall biosynthesis, photosystem efficiency, and hormonal regulation, amongst others, are critical for increased plant biomass accumulation [110,111,112]. Several annotated genes within QTL regions linked to biomass have been identified in the current study. We identified the *cellulose synthase A4* gene that is essential for cellulose production, a significant component of the plant cell wall [113]. Two *Expansins loci*, which are cell wall proteins that promote cell wall loosening and stress relaxation [114], and *cellulose synthase* protein, further support cell wall biosynthesis. Similarly, two loci encoding *Photosystem II–PSII proteins*, *D2* and *D1*, crucial for enhancing net CO2 assimilation rates with increases in biomass [115], were identified. The *Vigun07g005700* encodes a *PSII reaction centre protein K*, and *Vigun07g095600* encodes *NAD(P)H-quinone oxidoreductase subunit K*, both key players in photosynthesis and energy production. Furthermore, hormone regulation also significantly impacts biomass. The locus *Vigun03g160600* encodes *xyloglucan endotransglucosylase/hydrolase 28*, regulating cell wall growth [116]. *Vigun03g164100* encodes *auxin response factor 4*, which is essential for growth regulation [117]. Additional *auxin-responsive genes* include *Vigun01g175400*, *Vigun01g160800*, *Vigun01g161500*, and *Vigun01g161700*, the key to growth and developmental processes in many plants [118]. The *Vigun07g058400* encodes an *auxin response factor 18-like protein*, further influencing biomass accumulation. *Vigun07g086100*, encoding *heat shock protein 70*, aids in stress responses, indirectly supporting biomass under stress conditions (105,107). Regarding gibberellins, *Vigun07g037500* encodes a *gibberellin-regulated protein* essential for key developmental processes such as cell and plant elongation [119]. *Vigun07g038800* encodes a *bZIP transcription factor* involved in hormone signalling and stress responses, contributing to biomass production [120].

#### 4.2.4. Grain-Yield Genomic Loci and Candidate Genes

Grain yield is a critical trait in cowpea breeding programs, as in most crop programs, as it directly influences crop productivity and economic returns for farmers. In the present study, the QTLs on Vu01, Vu02, Vu05, Vu06, Vu07, and Vu09 are particularly important, due to their MEA effects, which indicate their stability and reliability across different P conditions. These stable QTLs are valuable for breeding programs targeting enhanced and consistent grain yield across P environments. The other QTLs, while important, may require further investigation due to their environmental sensitivity. There are limited comparable studies on QTL associated with grain yield in cultivated cowpea. One of the studies by Muchero et al. [106] mapped QTLs for grain yield under water-limited conditions. They reported five significant loci associated with grain yield on LGs 02, 05, 07, and 10 [106], which now correspond to Vu07, Vu08, Vu02, and Vu10, in the current cowpea chromosome numbering [40]. In our study, we identified a QTL on Vu02 named _MK18_CGyH2 (16,831,532 to 19,125,369 bp), which is located about 3.9 Mb upstream of the grain yield loci reported by Muchero et al. [106] on SNP 1_1150 (23,096,494 bp) on LG07–Vu02. Another region we identified, _ZR18_CGyH7 (36,313,019 to 36,337,518 bp), was distant from SNP 1_0589 on LG2–Vu07 (5,320,099 bp), as reported by Muchero et al. [106]. Additionally, the terminal region of the grain yield QTL _KW18_CGyH4* (2,220,918 to 2,418,796 bp) was located approximately 860 Kb upstream of the locus 2_06769 (3,278,892 bp), associated with pod dry weight—a proxy for grain yields, due to high positive correlations between them [79]. Moreover, the QTL on _KW18_CGyH9* (5,272,634 to 5,432,334 bp) overlapped with three significant positions for grain-yield DTI (2_23949, 5,346,101 bp), (2_23950, 5,347,304 bp) and (2_11952, 5,364,438 bp), respectively [78]. These results suggest that breeding for high grain yield can benefit from considering QTLs associated with different P conditions, thereby providing valuable targets for enhancing yield-related traits. There are few agreements among QTL studies focused on yield components in most crops, indicating that a larger number of alleles contribute to the observed variance [29]; such loci are often inconsistent and rarely reproducible over time [121], making them challenging to identify in different environments or populations [21].

The present study identified candidate genes associated with seed or grain production through their involvement in processes linked to seed development, maturation, and stress response during seed filling. On Vu09, *Vigun09g116600*, encoding *gibberellin 20 oxidase 2-like*, is involved in the biosynthesis of gibberellins. *Vigun09g126200*, which encodes a *nodulin MtN21/EamA-like* transporter family protein, is implicated in nutrient transport. Additionally, *Vigun01g167600* encodes a *35 kDa seed maturation protein of the LEA* family, which is essential for seed development [92]. Further, *Vigun01g173000* encodes the *flowering locus protein T*, a key regulator of flowering time, indicating the timing of flowering determines crop yield [94,122]. Locus *Vigun01g173200* encodes an *abscisic acid response element-binding factor 1 (ABF1)* protein required for seedling establishment in *Arabidopsis* during winter, regulating seed dormancy and germination [123]. *Vigun11g151800* encodes a *legumin-type B-like* protein, a storage protein vital for seed nutrition and quality. Other proteins involved in stress responses during seed development were identified: for instance, *Vigun11g122500*, which encodes *a LEA protein*, which is critical in protecting seed cells from desiccation [92]. Similarly, *Vigun09g014700*, encoding a *heat shock factor binding protein*, and *Vigun11g104600*, encoding a *DNAJ heat shock N-terminal domain-containing protein*, are involved in stress response mechanisms essential for maintaining seed viability under adverse conditions [124] and *Vigun11g206500*, which encodes a putative-*impaired sucrose induction protein*, is implicated in sucrose metabolism, a key process in seed filling and energy storage that directly impacts seed yield [125].

#### 4.2.5. QTLs for Grain PUE and PUpE Traits

Various measures of P-use efficiency are developed to analyse how crop plants absorb and use soil P [14,29,33,126]. The P-use efficiency traits assessed in this study were grain P-use efficiency (gPUE) and P-uptake efficiency (gPUpE) based on P accumulation in the grain [14,22]. QTLs associated with grain PUE under HP and LP conditions were identified. In the TV × IT population, one QTL was identified with a PVE of 12.9% under LP, whereas in the YA × 58 population, three QTLs for grain PUE were found under HP conditions with PVE values ranging from 4.5% to 26.9%. These results suggest that HP and LP conditions significantly influence grain P efficiency, with distinct QTLs playing pivotal roles. Similarly, in the TV × IT population, no significant QTLs were linked to grain PUpE under both soil-P conditions (Table S7). In the YA × 58 population, two QTLs were associated with grain PUpE under HP conditions, with PVE values ranging from 12.7% to 17.8%. Studies on cowpea marker-trait association for P efficiency are scarce, and comparisons were mostly made with related species and rice, where sufficient literature exists for response to P conditions, including reports of QTLs for P-associated traits under varying soil conditions in common bean [65,121,127], and soybean [128], where four major QTLs underlying P efficiency were identified. In rice, P-uptake-efficiency loci have been mapped [14,32], including the identification of QTLs for P-use and -uptake efficiency under an LP environment, where such loci collectively explained 54.5% and 42.1% of the phenotypic variation, respectively. In rice, the QTLs for P-use and -uptake efficiency coincided [32]. In another study under low- and normal-P conditions, 36 QTLs for P-efficiency traits, including grain P-use efficiency (gPUE) based on P accumulation in grains, were identified in rice [14].

Genes involved in P-use efficiency in plants typically relate to P acquisition, uptake, mobilization, and utilization [129,130]. Among the many genes in these QTL regions, some may be related to PUE in plants. For instance, *Vigun09g031800*, which encodes an *auxin efflux carrier*, might influence P use indirectly by affecting root development and, thus, the plant’s ability to explore the soil for P [131,132]. Similarly, *Vigun09g032600*, a *xyloglucan endotransglucosylase/hydrolase*, could play a role by remodelling cell walls, impacting root growth and P uptake [116]. Another gene, *Vigun09g035000*, encodes a *metalloendoproteinase 1-like protein* that could be involved in protein turnover and stress responses, potentially influencing P use under limiting conditions [133]. Additionally, *Vigun09g035700*, which codes for *cleavage and polyadenylation specificity factor 5*, is involved in mRNA processing, and may affect the expression of P-related genes, and *Vigun09g037400*, an *MYB transcription factor*, regulates various metabolic pathways, including those related to P acquisition and utilization [131]. Furthermore, five candidate genes with related functions to P-uptake efficiency by plants were identified on Vu09. The genes *Vigun09g005500 and Vigun09g005601* encode a *4-lactone oxidase family protein* and are involved in metabolic pathways that can affect P utilization. Similarly, *Vigun09g009200*, encoding *cytochrome c oxidase subunit 2*, plays a role in the electron transport chain, which is crucial for energy metabolism and indirectly impacts P-use efficiency. Additionally, *Vigun09g014700*, which encodes a *heat shock factor binding protein*, is involved in stress responses that can influence nutrient uptake and utilization. The fifth gene, *Vigun09g018100*, which encodes a *peroxidase superfamily protein*, can affect stress responses and nutrient assimilation.

For traits controlled by numerous small-effect QTLs, it is common to identify different sets of QTLs after repeating experiments or in different genetic backgrounds. One should not conclude that unreplicated QTLs are false, but rather reflect the power of detection of minor effect QTLs where a random portion is identified in any given experiment [134]. The significant QTLs identified in this study lay a foundation for further investigations into genetic mechanisms governing the traits assessed and marker-assisted selection aimed at the development of P-efficient varieties. The identification of QTLs on different chromosomes in both populations highlights the genetic complexity of traits like flowering, maturity, and grain yielding [79,80]. The environmental sensitivity of QTLs is evident in the differences in the number and effects of QTLs detected under HP and LP conditions, underscoring the need for environment-specific breeding strategies. Favourable alleles from both parental lines suggest allelic diversity that can be harnessed to enhance cowpea adaptability [77]. Differences in QTLs between populations highlight their distinct genetic architectures, suggesting the potential for combining multiple QTLs to improve adaptability and yield stability. QTLs identified under HP conditions suggest the potential for enhancing performance in P-rich soils, while those under LP indicate cowpea’s adaptability to P-deficient soils. This emphasizes the importance of targeting breeding efforts to specific environmental conditions. While this study represents a significant step in understanding the genetic basis of these important traits, future research should focus on fine-mapping QTL regions to identify precise candidate genes, investigating QTL–environment interactions, and utilizing advanced tools like genome-wide association studies (GWASs) and genomic prediction for more efficient breeding. Understanding both major- and minor-effect QTLs will enhance the genetic control of these traits and contribute to improving cowpea breeding for sustainable agriculture and food security.

## 5. Conclusions

There were substantial phenotypic variations among RILs under HP and LP soil conditions. The wide response to soil-P conditions indicates that the alleles conferring response to phenology, yield and P-use efficiency traits are quantitative. QTLs for days to flowering, maturity, biomass yield, grain yield, grain P-use and -uptake efficiency traits were mapped across nine chromosomes using the single- and multi-environment QTL analyses. Some QTL regions on Vu01, Vu03, Vu07, and Vu08 affecting flowering, maturity grain yield and P efficiency were consistently identified across multiple environments and P conditions. The stability of these QTLs across different P conditions is essential for developing resilient cowpea varieties capable of thriving in both P-rich and P-deficient environments. The present study identified annotated genes with functions related to the traits. Further fine mapping of these genomic regions is required for higher resolution and identification of a more concise set of candidate genes underlying the QTLs. Such information will allow the utilisation of the QTLs for the genetic improvement of cowpea for different edaphic environments where the crop is grown with little-to-no application of phosphate fertilizers by most smallholder farmers. Cowpea varieties with higher PUE and PUpE will require fewer P-based fertilisers, thereby reducing the cost of production on phosphate fertiliser inputs for growers.

## Figures and Tables

**Table 1:** QTLs identified for two phenology traits in two RIL populations under high and low soil-P conditions across environments in Nigeria.

RIL	Env	Trait	Soil P	QTl Name	Chr	Pos	Peak SNP	PT	LOD	L_SNP	R_SNP	PVE (%)	Effect	QTL Region
TV × IT	ZR.2017HP	Days to flowering	HP	_ZR17_CFtH8	8	31.8	2_53317	3.1	6.1	2_54699	2_01290	19.6	1.5	25.38–38.50
	ZR.2018HP		HP	_ZR18_CFtH8	8	41.1	2_01281	3.2	8.0	2_41633	2_01113	24.9	0.9	26.13–44.54
	MJ.2018HP		HP	_MJ18_CFtH1	1	56.3	2_24198	2.9	5.4	2_22354	2_07285	19.5	−2.4	51.48–58.60
	MJ.2018HP		HP	_MJ18_CFtH2 *	2	64.0	2_27943	2.7	46.2	2_27495	2_06055	31.7	10.0	63.5–64.5
	2017LP.ZR		LP	_ZR17_CFtL1	1	65.1	2_27671	3.3	3.5	2_41492	2_54893	11.5	−1.2	55.22–66.96
	2017LP.ZR		LP	_ZR17_CFtL8	8	31.8	2_51985	3.2	4.6	2_45772	2_00209	15.9	1.4	25.75–36.25
	2017LP.ZR		LP	_ZR17_CFtL2 *	2	68.0	1_1135	3.1	3.7	2_10955	2_46236	7.9	1.1	67.5–68.5
	2017LP.ZR		LP	_ZR17_CFtL3 *	3	52.0	2_28763	3.1	3.6	2_27392	2_50074	8.0	1.1	52.5–53.5
YA × 58	MK.2018HP		HP	_MK18_CFtH9	9	44.4	2_54431	3.1	3.3	2_21235	2_04825	16.6	1.4	31.50–47.91
	2017LP.ZR		LP	_ZR17_CFtL7	7	25.1	2_55072	3.1	3.4	2_51615	2_16942	17.3	1.4	14.67–67.66
TV × IT	ZR.2018HP	Days to maturity	HP	_ZR18_CMtH3	3	72.3	1_0718	3.1	3.4	2_38710	2_17221	10.7	−0.7	62.91–78.74
	ZR.2018HP		HP	_ZR18_CMtH8	8	41.1	2_01281	3.1	7.0	2_46238	2_01113	21.4	1.0	36.63–44.54
TV × IT	ZR.2017HP		HP	_ZR17_CMtH2 *	2	11.0	2_21892	2.8	2.9	2_10218	2_05606	4.3	0.6	10.5–12.5
	ZR.2017HP		HP	_ZR17_CMtH6.1 *	6	27.0	2_02446	2.8	9.7	2_00179	1_1042	16.8	−1.1	26.5–29.5
	ZR.2017HP		HP	_Z17_CMtH6.2 *	6	43.0	2_07337	2.8	4.9	1_0148	1_1020	7.5	0.8	42.5–43.5
	2018LP.MK		LP	_MK18_CMtL1 *	1	59.0	2_17448	3.0	3.3	2_01133	2_04568	10.4	0.4	58.5–59.5
	2018LP.MK		LP	_MK18_CMtL9 *	9	47.0	2_09944	3.0	3.1	1_1101	1_0892	9.8	0.4	46.5–47.5
YA × 58	2018LP.MJ		LP	_MJ18_CMtL9	9	13.7	2_14272	3.1	3.8	2_54962	2_21235	20.0	1.7	0.50–31.51
	2017LP.ZR		LP	_ZR17_CMtL8 *	8	83.0	2_55130	3.2	3.4	2_03348	2_55335	15.2	−0.9	82.5–84.5

QTLs are designated as follows: “C” to indicate cowpea, followed by the trait code, soil-P level (H = HP and L =LP), then followed by the chromosome number. QTL names are preceded with a subscript of the location and year of the trial, where ZR17 = 2017 Zaria trial, ZR18 = 2018 Zaria trial, and MJ18 = 2018 Minjibir trial. DTF = days to flowering, DTM = days to maturity, Chr = chromosome, Pos = genetic position in centimorgan, PT.LOD = permutation test LOD threshold, PVE = phenotypic variance explained, Effect = a positive value indicates the allele of the TVu-14676 or Yacine alleles are present, and a negative value indicates the allele of the IT84S-2246 or 58-77 alleles are present, depending on the RIL, Ft = flowering time, Mt = maturity time, HP = 60 kg/ha P_2_O_5_ applied, LP = no external P supplied to the soil. * QTL names with (*) indicate loci identified only by QTL IciMapping software, and those without (*) are identified by both Rqtl (version 1.70) and QTL IciMapping software (verson 4.2).

**Table 2:** QTLs identified for yield and grain P-efficiency traits in two RIL populations under high and low soil-P conditions across environments in Nigeria.

RIL	Env	Trait	Soil P	QTL Name	Chr	Pos	Peak SNP	PT	LOD	L_SNP	R_SNP	PVE (%)	Effect	QTL Region (CI)
TV × IT	ZR.2018HP	BYLD	HP	_ZR18_CByH3	3	43.74	2_23724	3.1	3.5	2_55371	2_23495	12.0	−10.2	40.75–50.50
TV × IT	MK.2018HP	BYLD	HP	_MK18_CByH1	1	37.99	2_49892	3.1	3.2	2_49686	2_16339	10.7	−9.0	28.97–57.10
TV × IT	KW.2018HP	BYLD	HP	_KW18_CByH7	7	7.26	2_53380	2.8	3.4	2_00227	2_47943	11.5	3.7	0.00–38.43
TV × IT	ZR.2018HP	BYLD	HP	_ZR18_CByH9 *	9	91.00	2_10684	3.0	3.7	1_0058	1_0167	10.3	−9.3	90.50–91.50
YA_58	KW.2018HP	BYLD	HP	_KW18_CByH4 *	4	12.00	2_54543	3.3	3.6	2_00188	2_50811	18.7	−13.1	10.50–12.50
TV × IT	MK.2018HP	GYLD	HP	_MK18_CGyH9	9	42.14	2_18613	2.9	4.0	2_54521	2_20569	13.2	93.5	38.77–59.55
TV × IT	2018LP.ZR	GYLD	LP	_ZR18_CGyL6	6	15.10	2_43166	2.9	3.7	2_48998	2_20013	13.6	17.5	13.98–31.08
YA_58	ZR.2018HP	GYLD	HP	_ZR18_CGyH9	9	8.51	1_0298	3.2	3.4	1_0519	2_39178	17.0	169.8	0.00–11.82
YA_58	MK.2018HP	GYLD	HP	_MK18_CGyH2	2	18.20	2_22304	3.2	7.2	2_54208	2_05606	32.5	257.8	12.20–19.68
YA_58	MJ.2018HP	GYLD	HP	_MJ18_CGyH1	1	59.78	2_38619	3.1	5.1	2_53926	2_05354	25.8	194.0	48.73–63.54
YA_58	KW.2018HP	GYLD	HP	_KW18_CGyH11	11	71.70	2_06469	3.1	3.3	2_50256	2_18440	17.1	128.8	56.18–111.72
YA_58	ZR.2018HP	GYLD	HP	_ZR18_CGyH7 *	7	126.00	2_14631	3.2	3.4	2_15327	2_37061	13.0	−143.0	125.5–127.5
YA_58	MK.2018HP	GYLD	HP	_MK18_CGyH3 *	3	79.00	2_55314	3.2	3.2	2_38936	2_19001	10.3	164.1	78.50–80.50
YA_58	KW.2018HP	GYLD	HP	_KW18_CGyH4 *	4	12.00	2_54543	3.2	3.6	2_00188	2_50811	18.0	−13.1	10.50–12.50
YA_58	KW.2018HP	GYLD	HP	_KW18_CGyH9 *	9	35.00	2_55390	3.2	3.2	2_37887	2_01864	14.9	−12.0	31.50–35.50
TV × IT	2018LP.ZR	gPUE	LP	_ZR18_CGpueL6	6	15.10	2_43166	2.7	3.3	2_50706	2_20013	12.7	12.9	13.23–31.08
YA_58	ZR.2018HP	gPUE	HP	_ZR18_CGpueH1 *	1	71.00	1_0910	3.0	3.2	2_05224	1_1013	4.5	−86.1	70.50–72.50
YA_58	ZR.2018HP	gPUE	HP	_ZR18_CGpueH9.1 *	9	9.00	2_16579	3.0	13.9	1_0298	2_52427	26.9	−210.5	8.50–10.50
YA_58	ZR.2018HP	gPUE	HP	_ZR18_CGpueH9.2 *	9	16.00	2_02382	3.0	6.9	2_54753	2_13277	10.8	135.7	15.50–16.50
YA_58	ZR.2018HP	gPUpE	HP	_ZR18_CGpupH9	9	8.51	2_20029	3.2	3.6	2_51098	2_39178	17.8	0.7	0.00–11.82
YA_58	ZR.2018HP	gPUpE	HP	_ZR18_CGpupH7 *	7	126.00	2_14631	3.1	3.2	2_15327	2_37061	12.7	−0.6	125.50–127.50

QTL are designated as follows: “C” to indicate cowpea, followed by the trait code, soil P (H = HP and L = LP), and then followed by the chromosome number. QTL names are preceded with a subscript of the year and location of the trial where ZR18 = 2018 Zaria trial, and MK18 = 2018 Mokwa trial. BYLD = biomass yield, GYLD = grain yield, GPUE = grain P-use efficiency, GPUpE = grain P-uptake efficiency. Chr = chromosome, Pos = genetic position in centimorgan, PT.LOD = permutation test LOD threshold, PVE = phenotypic variance explained, Effect = a positive value indicates the allele of the TVu-14676 or Yacine alleles are present, and a negative value indicates the allele of the IT84S-2246 or 58-77 alleles are present, depending on the RIL, By = biomass yield, Gy = grain yield, HP = 60 kg/ha P_2_O_5_ applied, and LP = no external P supplied to the soil. * QTL names with (*) indicate loci were identified only by QTL IciMapping, and those without (*) were identified by both Rqtl and QTL IciMapping software.

**Table 3:** QTLs for days to flowering, maturity and yield in RIL populations under high and low soil-P conditions across environments in Nigeria.

RIL	Trait	Soil P	QTL Name *	Chr	Pos	Peak SNP	Left Marker	Right Marker	PT	LOD	LOD (A)	LOD (AbyE)	PVE	PVE (A)	PVE (AbyE)	Add	A byE _01	A byE _02	A byE _03	A byE _04	A byE _05	QTL Region
TV × IT	DTF	HP	_ME_CFtH1	1	56	2_24198	2_33134	2_30425	4.7	7.3	7.2	0.1	27.3	11.4	16.0	0.4	−0.1	−0.4	0.8	−0.3	NA	55.5–56.5
		HP	_ME_CFtH8.1	8	32	2_53317	2_06417	2_03632	4.7	7.3	3.5	3.7	10.7	5.3	5.4	−0.3	−0.5	0.2	0.1	0.2	NA	31.5–32.5
		HP	_ME_CFtH8.2	8	41	2_01281	1_0040	2_55442	4.7	10.6	1.3	9.3	3.0	1.9	1.0	−0.2	0.2	0.0	−0.2	0.0	NA	40.5–41.5
		LP	_ME_CFtL1	1	65	2_45137	2_13572	2_07925	4.7	5.9	4.4	1.5	9.2	5.6	3.6	0.4	0.5	−0.2	−0.2	−0.2	NA	63.5–66
		LP	_ME_CFtL3	3	76	2_09795	2_01134	1_0900	4.7	5.8	3.7	2.1	5.8	4.5	1.3	0.3	0.2	0.1	−0.2	−0.1	NA	75.5–77.5
		LP	_ME_CFtL5	5	10	2_14678	2_07583	2_16147	4.7	4.9	4.1	0.8	8.0	5.0	3.0	−0.3	0.0	0.3	−0.4	0.1	NA	9.5–11.5
		LP	_ME_CFtL8	8	32	2_51985	2_06417	2_03632	4.7	8.2	4.4	3.8	11.7	5.5	6.2	−0.4	−0.6	0.1	0.4	0.1	NA	31.5–32.5
YA_58		LP	_ME_CFtL7	7	48	1_0525	2_11279	2_52128	5.0	5.1	0.9	4.2	13.7	3.1	10.6	−0.2	−0.6	0.2	0.2	0.2	NA	47.5–48.5
		LP	_ME_CFtL9	9	10	2_19358	2_52427	2_39178	5.0	5.2	3.0	2.2	18.4	10.6	7.8	−0.3	−0.3	0.3	−0.2	0.2	NA	9.5–11.5
TV × IT	DTM	HP	MECMtH3	3	72	1_0718	2_06373	1_0345	4.8	5.9	2.5	3.5	6.6	4.3	2.4	0.2	−0.1	0.0	0.2	−0.1	NA	71.5–72.5
		HP	MECMtH8	8	41	2_01281	1_0040	2_55442	4.8	9.9	1.4	8.5	4.5	2.5	2.0	−0.1	0.1	−0.2	−0.0	0.1	NA	40.5–41.5
TV × IT	GYLD	HP	MECGyH5	5	12	2_01573	2_00298	2_00867	5.1	5.3	3.0	2.2	6.0	4.9	1.0	11.6	−5.1	−5.0	0.1	9.4	0.5	11.5–12.5
		HP	MECGyH7	7	32	2_43413	2_41970	2_27708	5.1	5.5	3.5	2.0	6.9	5.6	1.3	12.2	−6.3	3.1	−3.0	10.2	−3.9	30.5–34.5
		HP	MECGyH9	9	42	2_18613	2_42491	2_43344	5.1	5.4	2.0	3.4	18.9	3.3	15.6	−9.4	7.1	8.4	1.7	−38.7	21.67	41.5–43.5
		LP	MECGyL6	6	14	2_48998	1_0933	2_05447	5.0	40.5	0.0	40.5	12,978.1	1.6	12,976.5	0.2	1.1	−23.6	−7.8	31.6	−1.3	13.5–14.5
YA_58		HP	MECGyH1	1	60	2_38619	2_06347	2_08207	5.5	8.6	5.4	3.1	12.5	8.4	4.1	−33.0	22.6	−16.2	19.1	−37.5	12.0	59.5–60.5
		HP	MECGyH2	2	18	2_22304	2_39619	2_22305	5.5	8.3	3.9	4.4	27.2	5.9	21.3	−28.0	29.4	25.3	−106.4	25.1	26.6	17.5–18.5
		HP	MECGyH7	7	126	2_14631	2_15327	2_37061	5.5	6.5	5.0	1.5	10.3	7.5	2.8	−31.0	24.1	−5.3	2.5	11.2	−32.6	125.5–127.5
		HP	MECGyH9	9	9	2_16579	1_0298	2_52427	5.5	6.5	3.2	3.3	8.8	4.8	3.9	−24.9	18.1	−33.2	−6.7	30.6	−8.9	8.5–9.5
TV × IT	gPUpE	LP	MECGpupL6.1	6	13	2_27869	2_24332	2_00562	3.4	13.2	0.1	13.0	1.9	1.1	0.9	0.1	−0.1	0.1	NA	NA	NA	12.5–13.5
		LP	MECGpupL6.2	6	15	2_43166	2_02969	2_47458	3.4	20.8	0.1	20.7	4.2	0.5	3.8	−0.1	0.2	−0.2	NA	NA	NA	14.5–15.5
YA_58		HP	MECGpupH1	1	71	1_0910	2_05224	1_1013	3.8	4.3	0.1	4.2	26.0	0.4	25.6	−0.0	0.2	−0.2	NA	NA	NA	69.5–71.5
		HP	MECGpupH9	9	9	2_16579	1_0298	2_52427	3.8	4.3	3.0	1.2	25.4	19.4	6.0	−0.2	0.1	−0.1	NA	NA	NA	8.5–9.5

QTLs are designated as follows: “C” to indicate cowpea, followed by the trait code, soil P (H = HP and L = LP where HP = 60 kg/ha P_2_O_5_ applied, LP = no external P supplied), and then followed by the chromosome number and sometimes with an ordered number when more than one QTL is detected in a single chromosome for a trait. “ME” preceding QTLs indicate multi-environment QTL. DTF = days to first flowering, DTM = days to maturity, Chr = chromosome, Pos = position in centimorgan, PT.LOD = permutation test LOD threshold, LOD = LOD scores for detecting QTLs with average effect across the environments and QEI effects, LOD (A) = LOD for QTLs with only average effects, LOD (AbyE) = QTLs with only QEI effects, PVE indicates phenotypic variation expressed by QTLs with average effect across the environments and QEI effects, PVE (A) indicates phenotypic variation expressed by QTLs with only average effects, PVE (AbyE) indicates phenotypic variation expressed by QTLs with QEI effects. Add indicates allele effects for which a positive value indicates the allele of the TVu-14676 or Yacine is present, and a negative value indicates the allele of the IT84S-2246 or 58-77 is present. * QTLs identified by IciMapping multi-environment (MET) functionality.

**Table 4:** Candidate genes associated with Ft and MT QTL regions under varying soil-P levels.

Trait	Locus Name	Chr	Start (BP)	End (BP)	Functional Annotation
FT	*Vigun08g080300*	Vu08	16,688,263	16,692,401	Zinc finger protein *CONSTANS-LIKE 1-like* (*COL1*) [*Glycine max*]
FT, MT	*Vigun08g116900*	Vu08	28,431,899	28,441,998	Zinc finger protein *CONSTANS-LIKE 9-like* (*COL9*) isoform X4 [*Glycine max*], B-box
FT	*Vigun08g119900*	Vu08	28,749,723	28,751,307	*Early flowering 4* (*ELF4-like 1*) protein
FT, MT	*Vigun08g124000*	Vu08	29,414,744	29,416,716	Zinc finger protein *CONSTANS-LIKE 16-like* (*COL16*) [Glycine max]
FT	*Vigun08g124100*	Vu08	29,426,933	29,428,985	*UDP-Glycosyltransferase superfamily protein* (*UGT87A2*)
FT, MT	*Vigun08g127400*	Vu08	29,776,355	29,778,144	Zinc finger protein *CONSTANS-like isoform X2* (*COL isoform X2*) [*Glycine max*]
FT, MT	*Vigun08g128600*	Vu08	29,870,661	29,873,299	*Putative, Snf1-related kinase interactor 1*
FT	*Vigun01g198701*	Vu01	37,535,304	37,544,023	*Flowering time control protein FCA-like isoform X1*
FT	*Vigun01g205500*	Vu01	38,123,633	38,129,571	*Light-sensor Protein kinase/phytochrome A* (*PHY1*)
FT	*Vigun01g227200*	Vu01	39,997,914	39,998,929	*Early flowering 4* (*ELF4-like 1*) protein
FT	*Vigun01g246100*	Vu01	41,443,163	41,444,980	*Kelch repeat F-box protein*
FT	*Vigun09g050600*	Vu09	4,991,501	4,996,844	*Phytochrome E* (*PHYE*)
FT	*Vigun07g025800*	Vu07	2,314,524	2,315,624	*LATE EMBRYOGENESIS ABUNDANT PROTEIN-25* (*LEA 25*)
FT	*Vigun07g046300*	Vu07	4,690,443	4,710,538	*EMBRYONIC FLOWER 2-like isoform X1*
FT	*Vigun07g046350*	Vu07	4,717,270	4,722,721	*EMBRYONIC FLOWER 2-like isoform X2*
FT	*Vigun07g059700*	Vu07	6,715,955	6,717,275	*Flowering locus protein T*
FT	*Vigun07g090150*	Vu07	14,187,820	14,188,269	*Flowering locus protein T*
FT	*Vigun07g106500*	Vu07	19,562,940	19,571,525	*Transcription factor jumonji domain protein* (*JmjC*)
FT	*Vigun07g116500*	Vu07	21,497,244	21,499,430	Zinc finger protein *CONSTANS-LIKE 13-like* (*COL13*) [*Glycine max*], B-box
FT	*Vigun07g133700*	Vu07	24,343,208	24,349,373	*Light-sensor Protein kinase//PHY1*
FT	*Vigun01g213600*	Vu01	38,754,573	38,757,589	*MADS-box* transcription factor family protein, (*MADS-box*)
FT	*Vigun01g248900*	Vu01	41,593,170	41,597,280	*MADS-box* transcription factor family protein *K-box*, *MADS-box*
FT	*Vigun07g034000*	Vu07	3,252,362	3,256,642	*MADS-box* transcription factor 6 [*Glycine max*], *K-box*, *MADS-box*
FT	*Vigun07g134900*	Vu07	24,504,608	24,506,390	*MADS-box* transcription factor family protein, *MADS-box*
FT	*Vigun07g135100*	Vu07	24,526,022	24,526,501	*MADS-box* transcription factor family protein, *MADS-box*
FT	*Vigun08g072700*	Vu08	12,094,658	12,099,310	*MADS-box* transcription factor family protein, *K-box*, *MADS-box*
FT	*Vigun08g096900*	Vu08	23,317,821	23,326,844	*MADS-box* transcription factor family protein, *MADS-box*
FT	*Vigun08g110400*	Vu08	27,428,213	27,443,026	*MADS-box* transcription factor 6 [*Glycine max*], *K-box*, *MADS-box*
FT	*Vigun09g059700*	Vu09	6,079,010	6,087,856	*MADS-box* transcription factor 6 [*Glycine max*], *K-box*, *MADS-box*
MT	*Vigun03g260900*	Vu03	42,765,664	42,770,048	Zinc finger protein *CONSTANS-LIKE 5-like* (*COL5*) [*Glycine max*], B-box
MT	*Vigun03g292500*	Vu03	47,756,399	47,757,106	*LEA-25* protein, seed maturation protein [*Glycine max*]
MT	*Vigun09g015300*	Vu09	1,120,677	1,124,820	*Flowering promoting factor 1* (*FPF1*)
MT	*Vigun09g015400*	Vu09	1,124,447	1,124,749	*Flowering promoting factor 1* (*FPF1*)
MT	*Vigun09g015500*	Vu09	1,134,327	1,134,939	*Flowering promoting factor 1* (*FPF1*)
MT	*Vigun09g037200*	Vu09	3,271,457	3,272,182	*ELF4-like 3 proteins*
MT	*Vigun09g050200*	Vu09	4,942,907	4,944,434	*MADS-box transcription factor family protein* (*MADS-box*)

FT is flowering time, MT is maturity time, BP is base pairs, Locus name is the name of the gene in the cowpea reference genome, Start is the beginning of the QTL region in bases, and End is the ending of the region in base pairs.

**Table 5:** Candidate genes associated with yield-related QTL regions under varying soil-P levels.

**Trait**	**Locus Name**	**Chr**	**Start (BP)**	**End (BP)**	**Functional Annotation**
BYLD	*Vigun03g152800*	Vu03	16,131,959	16,137,190	*Cellulose synthase* A4
BYLD	*Vigun01g164500*	Vu01	34,633,282	34,635,438	*Cell wall protein EXP2*
BYLD	*Vigun01g164600*	Vu01	34,639,109	34,640,295	*Cell wall protein EXP2*
BYLD	*Vigun07g092900*	Vu07	14,867,379	14,881,197	*Cellulose synthase family protein*
BYLD	*Vigun03g150850*	Vu03	15,830,990	15,831,584	L/M *photosystem II protein D2* [*Glycine max*]
BYLD	*Vigun07g095200*	Vu07	15,537,248	15,538,442	Photosystem II protein D1 [*Glycine max*], L/M
BYLD	*Vigun07g005700*	Vu07	451,603	452,013	Photosystem II reaction center protein K
BYLD	*Vigun07g095600*	Vu07	15,549,655	15,550,293	20 Kd subunit, *NAD*(*P*)*H-quinone oxidoreductase* subunit K
BYLD	*Vigun03g160600*	Vu03	17,742,874	17,745,514	*Xyloglucan endotransglucosylase*/*hydrolase* 28
BYLD	*Vigun03g164100*	Vu03	18,539,974	18,548,662	*Auxin response factor 4*
BYLD	*Vigun01g175400*	Vu01	35,668,742	35,669,758	*SAUR-like auxin-responsive protein family*
BYLD	*Vigun01g160800*	Vu01	34,269,522	34,269,899	*SAUR-like auxin-responsive protein family*
BYLD	*Vigun01g161500*	Vu01	34,334,180	34,335,025	*SAUR-like auxin-responsive protein family*
BYLD	*Vigun01g161700*	Vu01	34,345,146	34,345,826	*SAUR-like auxin-responsive protein family*
BYLD	*Vigun07g058400*	Vu07	6,436,308	6,438,943	*Auxin response factor 18-like* [*Glycine max*]
BYLD	*Vigun07g086100*	Vu07	13,029,458	13,032,760	*Heat shock protein 70*
BYLD	*Vigun07g037500*	Vu07	3,599,545	3,606,469	*Gibberellin-regulated family protein*
BYLD	*Vigun07g038800*	Vu07	3,759,715	3,762,378	*BZIP transcription factor family protein*
GYLD	*Vigun09g116600*	Vu09	25,552,438	25,555,314	*Gibberellin 20 oxidase 2-like* [*Glycine max*]
GYLD	*Vigun09g126200*	Vu09	27,978,054	27,981,909	*Nodulin MtN21/EamA-like transporter family protein*
GYLD	*Vigun01g167600*	Vu01	34,938,180	34,939,669	*LEA-3*,*35 kDa seed maturation protein* [*Glycine max*]
GYLD	*Vigun01g173000*	Vu01	35,503,295	35,505,086	*Flowering locus protein T*
GYLD	*Vigun01g173200*	Vu01	35,531,233	35,532,782	*Abscisic acid-responsive element-binding factor 1*
GYLD	*Vigun11g151800*	Vu11	36,193,653	36,196,371	*Legumin type B-like* [*Glycine max*], *plant*
GYLD	*Vigun11g122500*	Vu11	32,984,324	32,985,206	*Late embryogenesis abundant protein*
GYLD	*Vigun09g014700*	Vu09	1,095,336	1,098,074	*Heat shock factor binding protein*
GYLD	*Vigun11g104600*	Vu11	30,279,096	30,279,716	*DNAJ heat shock N-terminal domain-containing protein*
GYLD	*Vigun11g206500*	Vu11	40,299,672	40,304,889	*Putative*, *impaired sucrose induction protein*
gPUE	*Vigun09g031800*	Vu09	2,706,873	2,711,267	*Auxin efflux carrier family protein*
gPUE	*Vigun09g032600*	Vu09	2,787,176	2,788,825	*Xyloglucan endotransglucosylase/hydrolase family protein*
gPUE	*Vigun09g035000*	Vu09	3,077,522	3,079,525	*Catalytic domain*, *metalloendoproteinase 1-like* [*Glycine max*]
gPUE	*Vigun09g035700*	Vu09	3,154,228	3,156,321	*Cleavage and polyadenylation specificity factor 5*
gPUE	*Vigun09g037400*	Vu09	3,299,965	3,302,656	*MYB transcription factor*
gPUpE	*Vigun09g005500*	Vu09	389,058	391,978	*4-lactone oxidase family protein*, *type 2*, *D-arabinono-1*,*4-lactone oxidase*
gPUpE	*Vigun09g005601*	Vu09	401,462	408,749	*4-lactone oxidase family protein*, *type 2*, *D-arabinono-1*,*4-lactone oxidase*
gPUpE	*Vigun09g009200*	Vu09	724,257	728,095	*Cytochrome c oxidase subunit 2*
gPUpE	*Vigun09g014700*	Vu09	1,095,336	1,098,074	*Heat shock factor binding protein*
gPUpE	*Vigun09g018100*	Vu09	1,359,629	1,361,514	*Peroxidase superfamily protein*

BYLD is biomass yield, GYLD is grain yield, gPUE is grain PUE, gPUpE is grain PUpE, BP is base pairs, Locus name is the name of the gene in the cowpea reference genome, Start is the beginning of the QTL region in bases, and End is the ending of the region in base pairs.

## Data Availability

The original contributions presented in the study are included in the article/Supplementary Material, further inquiries can be directed to the corresponding author.

## Supplementary Materials

The following supporting information can be downloaded at: https://www.mdpi.com/article/10.3390/genes16010064/s1, Table S1. List of trait names with their abbreviation and definition, Table S2: Genetic map of the Yacine by 58-77 RIL population and the genotype dataset used for its construction. “A” and “B” represent alleles derived from Yacine and 58-77, respectively. Lowercase letters mean that the genotype calls were reversed based on the parental alleles; Table S3: iSelect SNP consensus genetic map new numbering for cowpea; Table S4: Descriptive statistics, and broad-sense heritability (H2) for phenology, yield and grain P-efficiency traits evaluated in two RILs under high and low soil-P environments. The RILs and the parent were grown in different environments of years and locations. * & ** represent significance at 5% and 1%, respectively; Table S5: Combined data descriptive statistics, and broad-sense heritability (H2) for phenology, yield and grain P-efficiency traits evaluated in two RILs under high and low soil-P environments. The RILs and the parent were grown in different environments of years and locations. * & ** represent significance at 5% and 1%, respectively; Table S6: Combined BLUP analysis for phenology, yield and grain P efficiency traits evaluated in two RIL populations under high and low P-soil P environments across locations; Table S7: Phenotypic correlation of HP vs. LP treatments of individual and combined environments; Table S8: Physical position (bp) and size of the QTL regions; Table S9: Functional annotations for cowpea gene models: columns highlighted in blue are those extracted from the Legume Information System, while those in white are those functional annotations provided by Phytozome; Table S10: Multi-environment QTLs with LOD scores below permutation threshold in RIL populations under high and low soil-P conditions accounting for high phenotypic variance of the traits; Table S11: Single-environment QTLs with LOD scores below permutation threshold in RIL populations under high and low soil-P conditions accounting for high phenotypic variance of the traits.

## Appendix A

QTL Mapping for Cowpea using rQTL.
