# Supplementary material for: Quantitative Trait Loci for Phenology, Yield, and Phosphorus Use Efficiency in Cowpea
Source: Genes (Basel). 2025 Jan 8;16(1):64. doi: 10.3390/genes16010064 (PMC11764512; doi:10.3390/genes16010064)
Supplement: Supplementary file 1 [file genes-16-00064-s001.zip › QTL Mapping for Cowpea using rQTL.pdf]

###QTL Mapping for Cowpea using rQTL

```
##Set working directory-Click session then choose a directory

R.version.string

getwd()

set.seed(3)

## Step 1.1 Install the packages that we will use for QTL mapping

install.packages("qtl")

install.packages("snow")

##Step 1.2 Load the packages in the session to be able to use them

library(qtl)

library(snow)

library(dplyr)


#Step 2.1 Prepare the input files- put together both pheno and genetic map as csv file

#Step 2.2 Load the data files and associate them with R Objects

#We will use genotype (geno) and phenotype (pheno) data

geno <- "Geno_file_name.csv"

pheno <- "Pheno_file_name.csv"


# We indicate to the program where the data is, what they mean, and what type of population we want.

data <- read.cross("csvsr",".", geno, pheno, crosstype="riself")


#Step 3.1 summary of the data file, verify that there are no errors, and all the data has been imported

summary(data)


#Below gives more detailed summary

nind(data) #Number of individuals

nchr(data) #Number of chromosomes

totmar(data) #Total number of markers

nmar(data) #Number of markers by chromosome
```

```
nphe(data) #Number of phenotypes

phenames(data) #names of the phenotypes


#Step 3.2 plot of the phenotypic data giving No of individuals classified with each phenotype

plotPheno(data, pheno.col = 2)


#Step 3.3 plot of genetic map which represent distribution of the SNP markers on chromosomes

plotMap(data)


#Step 3.4 plot of genotypes annotated for each marker e.g., see in chr 1 for the 1st 20 ind.

plotGeno(data, chr=1, ind=c(1:20), min.step=4)


#Step 5.0 To avoid interference caused by SNP markers that are located at the same genomic position, we use jittermap

data<-jittermap(data)


# Initial exploration ends here, start actual mapping


                    ####   SINGLE QTL analysis

###Step 6 In this step, hidden markov model is used to to calculate the probabilities of the true underlying genotype taking the rest into account

###It would be a quality control prior to the analysis to determine if there are poorly genotyped SNPs

##step=0 indicates the use of existing markers, it does not create pseudomarkers

data <- calc.genoprob(data,step=0)


###Step 7 We carried out the first proper QTL mapping using the EM algorithm

# (simple interval mapping-sim)

out.sim <-scanone(data, pheno.col = 2)


###We observe the results

summary(out.sim)
```

```
plot(out.sim,ylim=c(1,10), main="grain SIM")


##Step 8 Next, we perform the mapping using the Haley-Knott regression method
        # (Composite interval mapping)
out.cim <-scanone(data, pheno.col = 2, method="hk")



#We observe the results
summary(out.cim)
plot(out.cim,ylim=c(1,10), main="biomass CIM")


##Step 11 next, we are going to perform a thousand permutations to establish the significance threshold
operm <- scanone(data, pheno.col = 2, method="hk", n.perm=1000)


##We check the LOD values from permutation tests corresponding to sign of 1%, 5%, and 20%."
summary(operm, alpha=0.05)


###We chose the CIM because it delimits the QTL interval much better
##Step 10 We save the data obtained from the HK mapping in a csv file."
write.csv(out.cim, file = "grain_lp_kadawa18.csv", row.names = TRUE)


###Set the threshold at a 5% error
###We extract the markers with a LOD score higher than this threshold
region_qtl<-filter(out.cim) %>% filter(lod>3.16)
names(region_qtl) <- c("chr", "cM", "lod")
region_qtl


###Store output of significant of QTLs in csv file
write.csv(region_qtl, file = "biomass_sign_QTLs_kadawa18_qtl_CIM.csv", row.names = TRUE)
```

```
#Step 12 Identify significant QTL intervals (CI) using LOD drop

CI <- lodint(results = out.cim, chr = 5, drop = 1.5, expandtomarkers=TRUE)

CI



##Step 13 Make QTL model with the significant marker

data <- calc.genoprob(data,step=0)

qtl <- makeqtl(data, chr=c(5), pos=c(16.860511),what=c("prob"))


fitqtl <- fitqtl(data, pheno.col= 2, formula = y~Q1, qtl= qtl, method = "hk", get.ests = T)

summary(fitqtl)


#To know a marker name at a position on a chr

effectmarker <- find.marker(data, chr =5, pos = 38.49111)

effectmarker
```
